# Have Socioeconomic Inequalities in Tobacco Use in India Increased Over Time? Trends From the National Sample Surveys (2000–2012)

**DOI:** 10.1093/ntr/ntw092

**Published:** 2016-04-05

**Authors:** Nandita Bhan, Anup Karan, Swati Srivastava, Sakthivel Selvaraj, S. V. Subramanian, Christopher Millett

**Affiliations:** 1 ^1^ Department of Research, Public Health Foundation of India, New Delhi, India;; 2 ^2^ Indian Institute of Public Health Delhi, Gurgaon, India;; 3 ^3^ Department of Social and Behavioral Sciences, Harvard School of Public Health, Boston, MA;; 4 ^4^ Department of Primary Care and Public Health, Imperial College, London, London, UK

## Abstract

**Introduction:**

India has experienced marked sociocultural change, economic growth and industry promotion of tobacco products over the past decade. Little is known about the influence of these factors on socioeconomic patterning of tobacco use. This study examines trends in tobacco use by socioeconomic status (SES) in India between 2000 and 2012.

**Methods:**

We analyzed data in 2014 from nationally-representative repeated cross-sectional National Sample Surveys (NSS) in India for 1999–2000, 2004–2005 and 2011–2012 (*n* = 346 612 households). Prevalence and volume trends in cigarette, “bidi” and smokeless tobacco use were examined by household expenditure, educational attainment and caste/tribe status using Two-part model.

**Results:**

Prevalence of any tobacco use remained consistent in the poorest households (61.5% to 62.7%) and declined among the richest (43.8% to 36.8%) between 2000–2012. Bidi use declined across all groups (poorest: 26.3% to 16.8%, richest: 19.8% to 10.7%) while cigarette use increased (poorest: 1.2% to 1.3%, richest: 6.5% to 7.0%). Relative to educated and general caste households, between 2000 and 2012 cigarette use in illiterate households increased by 38% and among Scheduled Tribe households increased by 32%. Smokeless tobacco use increased for all households (poorest: 26.2% to 33.9%, richest: 11.4% to 13.5%, Scheduled Tribe: 31.1% to 34.8%, general caste: 13.6% to 18.5%), with greater increases among richer, more educated and general caste households.

**Conclusion:**

Marked SES patterning of tobacco use has persisted in India. Improving enforcement of tobacco control policies and monitoring comprehensive smoke-free legislations are needed to address this growing burden.

**Implications:**

We found “resilient” tobacco patterns in the last decade despite prevention interventions. SES continues to be inversely associated with tobacco products, with the exception of cigarettes. The declines in bidi use may be getting replaced by increase in cigarette use trends, especially among lower SES groups. The use of smokeless tobacco products has increased across all SES groups and the volume of smokeless tobacco use is not been declining despite a number of policies on tobacco use. This may be attributed to inadequate attention to chewed forms of tobacco in current policies, particularly to implementing pictoral warnings and regulating surrogate advertising. Evaluating the implementation of anti-tobacco policies and ensuring equity dimensions in interventions is urgently needed to address tobacco use inequalities.

## Introduction

India has the second highest number of tobacco users in the world with 229 million users behind China’s 311 million.^1^ This high tobacco use has implications for India’s chronic disease burden manifesting as cardiovascular diseases, lung and oral cancers and respiratory illnesses.^2^ Tobacco use in India is unique compared to other contexts for three reasons—diversity in forms of tobacco used (cigarette, “bidi” and smokeless forms), scope for increased use among growing socioeconomically mobile populations, and the role of multiple socioeconomic and cultural stratifiers (income, education, caste/tribe status, etc) as risk determinants.^3–6^

Several studies have shown inverse gradients in tobacco use in India (with the exception of cigarette use) with generally greater use among poor, less educated and disadvantaged caste and tribe groups.^4,5,7–10^ The noted patterns may be attributed to differences in the penetration of tobacco control policies and variations in awareness about the health consequences of tobacco use. Tobacco use disparities have also been linked to differentials in uptake among adolescents, type and quality of products used and patterns in the cessation of use, with education, social norms, and cultural practices being important drivers.^8,9^

Little is known about changes in tobacco use over time by socioeconomic status (SES) in India. This evidence is important from three perspectives. *First*, economic growth, rising incomes and urbanization have increased access to tobacco products, for example, cigarettes are becoming more affordable across social strata. This may manifest in earlier initiation and access to tobacco products, which could vary by income or education.^6,7,11,12^ *Second*, increases in innovative marketing, packaging and promotion of tobacco products may differentially impact vulnerable populations. For instance, tribal populations may respond to innovative marketing by switching from traditional tobacco products (eg, snuff, hookah, kimam) to mass-market forms of tobacco (eg, bidis and cigarettes).^13,14^ *Finally*, in other contexts, nearly half of premature mortality between SES groups has been attributed to tobacco use.^15^ In India, where multiple forms of tobacco exist, social patterning of tobacco will be an important driver of inequalities in morbidity and mortality from noncommunicable diseases (NCDs) going forward. Tracking changes in the socioeconomic inequalities in tobacco use may aid in understanding the directions of this change, thereby identifying gaps in existing interventions in order to avert future disease burdens.

This study was conducted to assess the direction of trends in socioeconomic inequalities in tobacco use in India since 2000. Using nationally representative household level data, we examined changing trajectories of socioeconomic patterning in the prevalence and volume of bidi, cigarette and smokeless tobacco use in the country.

## Methods

### Data and Study Setting

We utilized data from three waves of the Consumer Expenditure Surveys, conducted by the Indian National Sample Survey Organization (NSSO)^16^ in 1999–2000, 2004–2005 and 2011–2012. The NSSO compiles nationally-representative repeated cross-sectional surveys on household consumption, expenditures and socioeconomic dynamics. In this analysis conducted in 2014, we used data from 346 616 households (1999–2000: 120 310; 2004–2005: 124 644 and 2011–2012: 101 662) from villages and urban blocks, sampled through stratified multi-stage sampling. Full details on the Consumer Expenditure Surveys can be accessed through website of the NSSO.^16^

Survey data were collected through face-to-face interviews with the head of the household. In case of his/her nonavailability, information was collected from any knowledgeable member of the household. Detailed data on consumption, including more than 350 food and non-food items, are available in the surveys. All data are available at the household level with information on individual members unavailable. Informed consent was obtained by the survey and identifiers were not available in the data.

### Variables

Main outcomes in the analysis were any household tobacco use and volume of tobacco use (in number of sticks for cigarette/bidi and grams for smokeless use) based on purchases within a 30-day recall period. The National Sample Survey collects data on tobacco use at the household level only. In India, tobacco is consumed in multiple forms including bidi, cigarette, leaf tobacco, snuff, hookah tobacco, cheroot, zarda (flavored tobacco blending tobacco leaves, sweeteners and other compounds), kimam (chewing tobacco used in betel leaves) and surti (dried tobacco leaves consumed with lime) and other tobacco products. We focused the analysis on three key tobacco products used in India (cigarettes, bidis and smokeless). These three products comprised nearly 97% of all tobacco-consuming households.^9^ Smokeless tobacco included snuff, zarda, kimam and surti, and the survey definitions of smokeless tobacco have remained constant over time. Given the possibility of use of multiple tobacco products in the same household or even by the same consumer, we analyzed trends in exclusive use of tobacco products separately from use of multiple or any tobacco product.

Three key household-level socioeconomic stratifiers were considered—expenditure quartiles, education and caste/tribe status. Expenditure quartiles provide the best proxy for income or living standards of households in the Indian context and are used frequently given large populations in non-formal employment.^17,18^ Consumption expenditure was classified by quartiles with Q1 representing the poorest 25% households and Q4 representing the richest 25% households. For ascertaining the highest educational attainment for the household, information on highest year of schooling for any household member was used to classify households as “illiterate households,” “households with at least one primary schooled member,” “households with at least one middle schooled member,” “households with at least one high schooled member,” and “households with at least one graduate.” Self-reported household caste or tribe affiliation was classified as “general,” Scheduled Caste (SC), Scheduled Tribe (ST), and “Other Backward Classes” (OBC). SCs, STs, and OBCs are “special groups” identified by the Indian government for affirmative action in education and other development policy, with general castes including the remaining population.

We adjusted for household size, mean age, gender composition, employment status (regular/salaried, self-employed agricultural, self-employed non-agricultural, casual laborers and others), religion (Hindu, Muslim, Christian and others) and area of residence (rural or urban) as covariates in the analysis.

### Analysis

Prevalence of exclusive cigarette, bidi and smokeless tobacco use were estimated along with use of any and multiple tobacco products by SES for the three survey rounds. We examined trends in consumption among households that reported prevalence and volume of tobacco use. Two-Part-Models were used to estimate trends in volume adjusted for potential selection bias from non-reporting of outcome by non-users.^17,18^ This approach also corrected for skewness in the distribution of tobacco use in the sample.

Two-Part-Models is estimated in two stages: first part (Part-I) is estimated as logistic regression by using full sample with dummy dependent variables having values “0” or “1.” This provides estimates on the probability of positive outcome. The second part (Part-II) is estimated as semi-log regression using the sample with only positive outcomes as continuous variable. Using the Two-Part-Models we estimated (1) the probability of households consuming tobacco (equation 1.1) and (2) the volume of consumption, conditional on reported tobacco use (equation 1.2). To examine the trends in prevalence and volume of consumption across our main predictors (wealth quintile, caste and education groups), we estimated interactions between the three SES categories and survey years using pooled data. We also adjusted the results for other SES features as outlined in the above paragraphs and used state level fixed effects to control state-specific characteristics.

$$\begin{array}{l} \log it(Y_{ijt})=\alpha +d_{t}+\sum_{q=1}^{3}Q_{q}\beta_{1q}+\sum_{e=1}^{4}E_{e}\beta_{2}{}_{e}+\sum_{c=1}^{3}C_{c}\beta_{3}{}_{c}\\ +\sum_{t=2}^{3}\sum_{q=1}^{3}Q_{qt}.d_{t}.\beta_{1qt}+\sum_{t=2}^{3}\sum_{e=1}^{4}E_{et}.d_{t}.\beta_{2}{}_{et}\\ +\sum_{t=2}^{3}\sum_{c=1}^{3}C_{ct}.d_{t}.\beta_{3}{}_{ct}+\beta_{4}X_{ijt}+\eta_{j}+\varepsilon_{it} \end{array}$$ (1.1)

$$\begin{array}{l} \log(Y_{ijt}|Y_{ijt}>0)=\alpha +d_{t}+\sum_{q=1}^{4}Q_{q}\lambda_{1q}+\sum_{e=1}^{4}E_{e}\lambda_{2}{}_{e}+\sum_{c=1}^{3}C_{c}\lambda_{3}{}_{c}\\ +\sum_{t=2}^{3}\sum_{q=1}^{4}Q_{qt}.d_{t}.\lambda_{1qt}+\sum_{t=2}^{3}\sum_{e=1}^{4}E_{et}.d_{t}.\lambda_{2}{}_{et}\\ +\sum_{t=2}^{3}\sum_{c=1}^{3}C_{ct}.d_{t}.\lambda_{3}{}_{ct}+\lambda_{4}X_{ijt}+\nu_{j}+\mu_{it} \end{array}$$ (1.2)

Where *Y*_*it*_ = tobacco product for household “*i*” at time “*t*,” *d*_*t*_ = time dummy (*t*_1_ = 2005, *t*_2_ = 2012), “Q1”–“Q3” are three expenditure quartiles (with reference: richest 25%), “E1”–“E4” are four education groups (with reference: graduate and above) and “C1”–“C4” are three caste groups (with reference: other). “Q_*it*._ *d*_*t*_” are interaction terms between expenditure quartiles and time periods. Similarly, “E_*et*._ *d*_*t*_” and “C_*it*._ *d*_*t*_” are interaction terms between education and caste groups and time periods, respectively. The interaction terms estimate relative changes in tobacco use for disadvantaged households relative to the most advantaged households; the constant “α” represents tobacco use for the richest, graduate and above and other caste in the reference year (2000). “ε_*it*_” (“μ_*it*_”) represents usual error term while η_*j*_ (ν_*j*_) is an additional error term representing fixed-effects in the respective equations. All other covariates are represented by the vector X.

equations 1.1 and 1.2 provide not only estimates of the interaction terms which represent changes in tobacco use (prevalence and volume consumption, respectively) in 2005 and 2012 with the base-year reference of 2000 but also the differences of the estimates of the interaction terms between 2005 and 2012 represent changes in prevalence and volume consumption between 2005 and 2012.

For any and multiple tobacco products, only trends by prevalence were estimated due to differences in reporting units for volume estimates. All analyses were conducted using STATA/ic v.12.1.

## Results

### Prevalence of Tobacco Use by Socioeconomic Group

**Figure 1.**
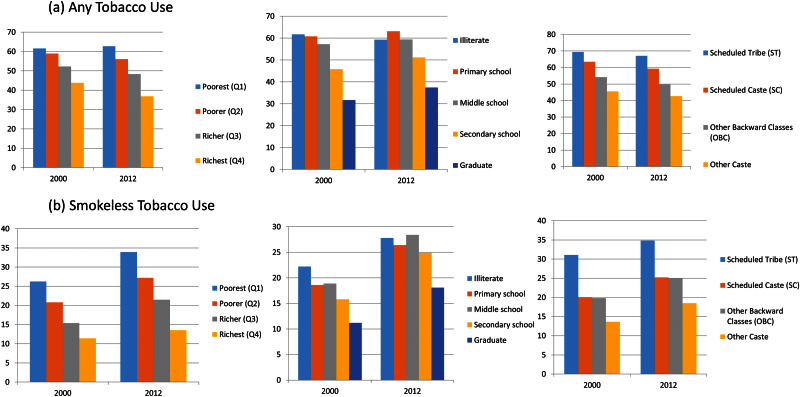
Socioeconomic inequalities in tobacco (%) over time (a) any tobacco use and (b) smokeless tobacco use by household consumption expenditure, schooling and caste/tribe status.

Table 1 reports use (%) of exclusive bidi, cigarette and smokeless tobacco by expenditure quartiles, education and caste/tribe and Supplementary Table 1 reports use of any and multiple tobacco products for the three survey periods. In 2012, 62.7% (95% confidence interval [CI]: 62.1,63.3) of households in quartile 1 (poorest) compared to 36.8% (95% CI: 36.3,37.4) in quartile 4 (richest) reported using any tobacco product (Figure 1). Nine percent (95% CI: 8.5,9.6) of households without educated members reported multiple tobacco use compared to 5.2% (95% CI:5.0,5.4) of households with at least one graduate. 59.2% (95% CI: 58.4,59.9) of SC households compared to 67.1% (95% CI: 66.4,67.9) of ST households reported any tobacco use in 2012.

**Table 1.** Prevalence (%) of Exclusive Cigarette, Bidi and Smokeless Tobacco Use Across Three National Sample Surveys (1999–2000, 2004–2005 and 2011–2012) by Consumption Expenditure, Educational Attainment and Caste/Tribe Status of Households

SES variables	*N* (%)	Bidi use			Cigarette use			Smokeless use		
		1999–2000	2004–2005	2011–2012	1999–2000	2004–2005	2011–2012	1999–2000	2004–2005	2011–2012
Household consumption expenditure quartiles										
Poorest quartile (Q1)	75 918 (21.9)	26.3 (25.75–26.82)	18.4 (17.95–18.89)	16.8 (16.33–17.29)	1.2 (1.04–1.30)	0.9 (0.79–1.02)	1.3 (1.13–1.42)	26.2 (25.68–26.75)	25.4 (24.87–25.91)	33.9 (33.32–34.54)
Poorer |quartile (Q2)	79 003 (22.8)	27.8 (27.31–28.35)	23.7 (23.21–24.19)	17.3 (16.78–17.77)	2.4 (2.23–2.59)	2.3 (2.12–2.47)	2.8 (2.62–3.05)	20.8 (20.35–21.30)	24.6 (24.14–25.14)	27.2 (26.61–27.77)
Richer quartile (Q3)	86 538 (24.9)	25.9 (25.50–26.49)	22.8 (22.32–23.26)	14.9 (14.55–15.43)	3.7 (3.47–3.89)	3.1 (2.94–3.33)	4.2 (3.99–4.49)	15.4 (15.03–15.85)	21.9 (21.49–22.41)	21.5 (21.04–22.05)
Richest quartile (Q4)	105 155 (30.3)	19.8 (19.38–20.19)	17.6 (17.21–17.97)	10.7 (10.32–11.01)	6.5 (6.24–6.75)	5.3 (5.09–5.53)	7.0 (6.77–7.35)	11.4 (11.11–11.76)	17.4 (17.01–17.77)	13.5 (13.11–13.88)
Highest educational attainment of households										
Households with illiterate members only	109 391 (31.6)	30.1 (29.64–30.65)	25.7 (25.41–26.08)	21.0 (20.29–21.78)	1.3 (1.15–1.40)	1.7 (1.63–1.83)	1.4 (1.15–1.58)	22.2 (21.73–22.64)	25.1 (24.74–25.39)	27.8 (26.99–28.63)
Households with **one** primary schooled member	47 415 (13.7)	31.9 (31.25–32.67)	17.4 (16.92–17.94)	23.9 (23.02–24.74)	2.1 (1.91–2.34)	3.6 (3.33–3.82)	1.5 (1.28–1.77)	18.6 (18.01–19.19)	21.2 (20.71–21.81)	26.4 (25.53–27.30)
Households with **one** middle schooled member	59 549 (17.2)	26.9 (26.38–27.47)	13.3 (12.78–13.83)	16.9 (16.45–17.54)	3.4 (3.15–3.59)	4.3 (4.01–4.64)	2.9 (2.71–3.20)	18.9 (18.46–19.42)	19.9 (19.30–20.54)	28.4 (27.79–29.11)
Households with **one** higher secondary schooled member	48 155 (13.9)	18.4 (17.95–18.89)	9.3 (8.15–10.49)	13.8 (13.28–14.26)	5.4 (5.13–5.68)	6.9 (5.91–7.94)	4.7 (4.41–5.01)	15.8 (15.41–16.28)	12.5 (11.19–13.87)	24.9 (24.33–25.55)
Households with **one** graduate	82 104 (23.7)	8.0 (7.67–8.43)	5.9 (5.61–6.28)	7.9 (7.74–8.25)	8.3 (7.88–8.66)	6.5 (6.15–6.84)	6.0 (5.81–6.25)	11.2 (10.77–11.66)	12.9 (12.45–13.39)	18.1 (17.74–18.47)
Household caste/tribe										
Scheduled Tribe (ST)	43 364 (12.5)	26.4 (25.65–27.15)	21.4 (20.76–22.02)	16.3 (15.67–16.91)	1.3 (1.11–1.49)	1.3 (1.15–1.50)	1.9 (1.76–2.23)	31.1 (30.37–31.94)	36.2 (35.52–36.99)	34.8 (34.04–35.64)
Scheduled Caste (SC)	54 502 (15.7)	32.7 (32.05–33.39)	26.7 (26.13–27.35)	20.8 (20.12–21.39)	1.9 (1.73–2.12)	1.7 (1.57–1.93)	2.8 (2.53–3.05)	19.9 (19.35–20.49)	23.3 (22.69–23.85)	25.2 (24.54–25.89)
Other Backward Class (OBC)	124 539 (35.9)	23.6 (23.18–24.03)	19.5 (19.13–19.85)	13.2 (12.92–13.58)	3.4 (3.19–3.55)	2.8 (2.65–2.95)	4.2 (4.03–4.42)	19.8 (19.28–20.07)	23.1 (22.74–23.51)	25.0 (24.57–25.42)
Other Caste (General)	124 209 (35.8)	21.9 (21.53–22.25)	18.0 (17.65–18.39)	13.2 (12.84–13.58)	4.8 (4.63–5.00)	4.2 (4.04–4.42)	4.6 (4.33–4.79)	13.6 (13.25–13.86)	16.9 (16.52–17.24)	18.5 (18.11–18.95)
Total	346 614	24.9 (24.73–25.22)	20.6 (20.40–20.85)	14.9 (14.72–15.16)	3.4 (3.34–3.54)	2.9 (2.82–3.01)	3.8 (3.73–3.97)	18.5 (18.27–18.71)	22.3 (22.11–22.57)	24.0 (23.78–24.31)

SES = socioeconomic status. Number of observations in each quartile group are unweighted numbers and hence may not be exactly 25% in each group.

**Figure 2.**
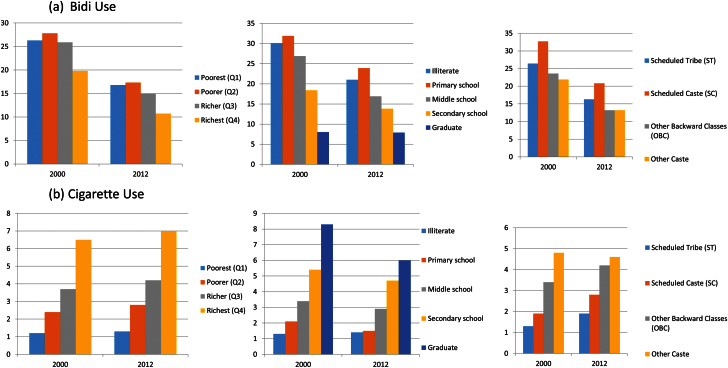
Socioeconomic inequalities in tobacco (%) over time (a) bidi use and (b) cigarette use by household consumption expenditure, schooling and caste/tribe status.

In 2012, 16.8% (95% CI: 16.3,17.3) of the poorest households compared to 10.7% (95% CI: 10.3,11.0) of the richest reported exclusive bidi use (Table 1 and Figure 2). Bidi consumption was 7.9% (95% CI: 7.7,8.3) among households with one graduate member compared to 21% (95% CI: 20.3,21.8) among households with illiterate members only. Cigarette use was 7% (95% CI: 6.8,7.3) among richest households compared to 1.3% (95% CI: 1.1,1.4) among the poorest households; and 6% (95% CI: 5.8,6.2) among households with a graduate compared to 1.4% (95% CI: 1.1,1.6) among households with illiterate members only (Table 1 and Figure 2). In 2012, 33.9% (95% CI: 33.3,34.5) of the poorest households reported smokeless tobacco use compared to 13.5% (95% CI: 13.1,13.9) of the richest households (Table 1 and Figure 1). About 27.8% (95% CI: 26.9,28.6) of households without educated members reported smokeless tobacco use compared to 18.1% (95% CI: 17.7,18.5) of households with a graduate member.

### Trend Analysis of Tobacco Products

#### Trends in Bidi Use

Among the reference base category (richest 25%, general caste households with at least one graduate member), bidi use declined by 13% during 2000–2005 and by 9% during 2000–2012 as indicated by the odds ratios (*OR*s) of time dummies “t1” and “t2.” This implies a 4% increase in bidi use between 2005 and 2012 across all households. Among bidi smokers, the volume of bidi use declined by 26% during 2000–2012, with most of the effective decline (approximately 23.5%) during 2005–2012 (Table 2, Supplementary Table 3). Compared to the reference base category, bidi use among the poorest 25% of households declined 14% faster during 2000–2005, but increased by 9% during 2000–2012. This implies that compared to the richest 25%, bidi use among the poorest 25% increased by 23% during 2005–2012. Similarly, bidi use declined by 36%, 12%, and 17% among illiterate, SC and ST households, respectively. Among bidi smokers, the volume of use decreased 5% faster among SC households but increased by 9% among ST households, relative to the reference base category. These results indicate that bidi use declined among SC and ST households during 2005–2012, but volume of use increased by 8% among ST households in the same period.

**Table 2.** Results From Pooled Two-part Models (TPM) for Prevalence and Volume of Exclusive Cigarette, Bidi and Smokeless Use by Consumption Expenditure, Educational Attainment and Caste/Tribe Status of Households (2000–2012)

	Bidi		Cigarette use		Smokeless	
	Part I: *OR*s (*SE*)	Part-II: log (volume) (*SE*)	Part I: *OR* (*SE*)	Part-II: log (volume)	Part I: *OR* (*SE*)	Part-II: log (volume)
*t*_1_ (2005)	0.87** (0.04)	−0.025 (0.03)	0.81*** (0.03)	−0.16*** (0.03)	1.49*** (0.06)	2.58*** (0.07)
*t*_2_ (2012)	0.91 (0.04)	−0.26*** (0.03)	0.74*** (0.04)	−0.37*** (0.04)	1.66*** (0.07)	2.41*** (0.08)
Q_1_ (poorest 25%)	1.58*** (0.04)	−0.23*** (0.01)	0.25*** (0.01)	−0.74*** (0.04)	1.40*** (0.04)	−1.03*** (0.04)
*t*_1__Q_1_ (interaction: 2005 **×** poorest 25%)	0.86*** (0.03)	−0.04* (0.02)	0.79** (0.07)	0.07 (0.06)	0.86*** (0.03)	0.80*** (0.06)
*t*_2__Q_1_ (interaction: 2012 **×** poorest 25%)	1.09* (0.04)	−0.18*** (0.02)	0.97 (0.07)	−0.12* (0.05)	1.11** (0.04)	0.95*** (0.06)
Q_2_ (poorer 25%)	1.50*** (0.03)	−0.11*** (0.01)	0.43***(0.02)	−0.46*** (0.03)	1.33*** (0.03)	−0.69*** (0.04)
*t*_1__Q_2_ (interaction: 2005 **×** poorer 25%)	1.01 (0.03)	−0.039* (0.02)	0.89 (0.05)	−0.05 (0.04)	0.92** (0.03)	0.59*** (0.06)
*t*_2__Q_2_ (interaction: 2012 **×** poorer 25%)	1.07 (0.04)	−0.07** (0.02)	1.05 (0.06)	−0.09** (0.04)	1.05 (0.04)	0.71*** (0.06)
Illiterate households (households with illiterate members only)	4.49*** (0.16)	0.13*** (0.02)	0.35*** (0.02)	−0.27*** (0.04)	1.45*** (0.05)	−0.27*** (0.05)
*t*_1__illiterate households (interaction: 2005 **×** illiterate households)	0.82*** (0.04)	−0.02 (0.03)	1.70*** (0.11)	−0.01 (0.05)	1.05 (0.04)	0.45*** (0.07)
*t*_2__illiterate households (interaction: 2012 **×** illiterate households)	0.64*** (0.03)	0.004 (0.03)	1.38*** (0.12)	−0.01 (0.07)	0.94 (0.04)	0.37*** (0.07)
Households with **one** primary schooled member	4.16*** (0.15)	0.11*** (0.02)	0.51*** (0.03)	−0.27*** (0.04)	1.47*** (0.05)	0.06 (0.06)
*t*_1__households with **one** primary schooled member (interaction: 2005 **×** household with primary schooled member)	0.62*** (0.03)	−0.08** (0.03)	1.65*** (0.11)	0.02 (0.05)	0.94 (0.04)	0.08 (0.08)
*t*_2__households with **one** primary schooled member (interaction: 2012 **×** households with primary schooled member)	0.64*** (0.03)	0.04 (0.03)	1.22* (0.10)	0.02 (0.06)	0.95 (0.05)	−0.09 (0.08)
SC households	1.63*** (0.04)	0.02 (0.01)	0.89* (0.04)	−0.15*** (0.04)	1.23*** (0.03)	−0.04 (0.04)
*t*_1__SC households (interaction: 2005 **×** SC households)	0.97 (0.03)	−0.007 (0.02)	0.94 (0.07)	0.11* (0.05)	0.99 (0.04)	−0.09 (0.06)
*t*_2__SC households (interaction: 2012 **×** SC households)	0.88** (0.03)	−0.05** (0.02)	1.14 (0.08)	0.02 (0.05)	0.93 (0.04)	0.03 (0.07)
ST households	1.30*** (0.04)	−0.12***(0.01)	0.97 (0.05)	−0.39*** (0.04)	1.62*** (0.04)	−0.44*** (0.04)
*t*_1__ST households (interaction: 2005 **×** ST households)	1.01 (0.04)	0.009 (0.02)	1.09 (0.07)	0.27*** (0.04)	0.85*** (0.03)	0.25*** (0.06)
*t*_2__ST households (interaction: 2012 **×** ST households)	0.83*** (0.03)	0.09*** (0.02)	1.32*** (0.08)	0.63*** (0.04)	0.69*** (0.03)	0.28*** (0.06)
Constant	0.002 (0.00012)	5.96 (0.04)	0.34 (0.03)	5.51 (0.07)	0.004 (0.00036)	3.17 (0.17)
Observations	346 614	87 787	346 614	28 938	346 614	69 102
*R* ^2^ (Pseudo *R* ^2^ for the *OR*s)	0.17	0.27	0.15	0.25	0.18	0.40

*OR* = odds ratio; SC = Scheduled Caste; SES = socioeconomic status; ST = Scheduled Tribe. Trend estimates allow for comparisons across time through *t*_1_ and *t*_2_ (indicators for time 1 and time 2 for reference group) and *t*_1_*SES and *t*_2_*SES (indicators of change from time 1 and time 2 for specific SES group—eg, poorest 20% households; reference: richest quartile, households with at least one graduate member and Other/general caste households in 2000). Figures in the second row for each indictor are standard errors (*SE*s).

*P < .05; **P < .001, ***P < .0001.

#### Trends in Cigarette Use

Cigarette use declined by 19% during 2000–2005 and by 26% during 2000–2012 among reference base category, implying a 7% decline during 2005–2012. Among cigarette smokers in the reference base category, volume of use declined by 16% during 2000–2005 and by 37% during 2000–2012, implying a net decline of 26% during 2005–2012. In comparison, cigarette use among the poorest 25% of households declined faster (21%) during 2000–2005, but the decline was not significant for 2000–2012. This implies that during 2005–2012, volume of cigarette use increased by 18%. Cigarette use among illiterate households increased by 70% (2000–2005) and 38% (2000–2012), implying a decline of 32% during 2005–2012. Cigarette use increased by 32% in ST households during 2000–2012 and by 23% during 2005–2012, while volume of use increased by 27% (2000–2005) and 63% (2000–2012) in ST households relative to the reference base category.

#### Trends in Smokeless Tobacco Use

Among reference base category, smokeless tobacco use increased by 49% during 2000–2005 and by 66% during 2000–2012, implying a 17% net increased during 2005–2012. Among smokeless tobacco users in the reference base category, substantial increases in volume were noted of nearly 258% during 2000–2005 and 241% during 2000–2012. In comparison, smokeless tobacco use declined by 24% during 2000–2005 and increased by 11% during 2000–2012 among the poorest 25%. The net increase during 2005–2012 was 35%. Increase in smokeless tobacco use during 2000–2012 among illiterate households were similar to the reference base category. ST households reported a decline of 15% during 2000–2005 and of 31% during 2000–2012. Among users, volume of smokeless tobacco use in ST households declined by 75% during 2000–2005 and 72% during 2000–2012. A marginal decline of 3% was noted in the volume of smokeless tobacco consumed among user households, but this was not statistically significant.

#### Trends in Any and Multiple Tobacco Use

Among reference base category, any tobacco use increased by 6% during 2000–2005 and by 17% during 2000–2012 (Supplementary Table 2). In comparison, any tobacco use declined by 28% during 2000–2005 and increased by 18% during 2000–2012 for the poorest 25%. Use of multiple tobacco products increased by 41% during 2000–2005 and by 49% during 2000–2012 among reference base category. In comparison, use of multiple tobacco products increased by 16% during 2000–2012 among the poorest 25%. Use of multiple tobacco products increased by 29% among illiterate households during 2000–2005 and by 17% among ST households (17%) during 2000–2012.

## Discussion

Our findings show that SES patterns have persisted over the last decade in India with inverse relationships between measures of socioeconomic disadvantage and tobacco use (except for cigarettes). In addition, three salient findings emerge. *First*, we found a sharp decline (9%–10%) in bidi use across all SES populations. *Second*, smokeless tobacco use has increased over the last decade across all socioeconomic groups. And finally, we found some evidence of increased uptake of cigarettes among lower SES groups, even as overall cigarette use has remained largely unchanged.

Tobacco prevention and control policies in India have largely focused on awareness and behavior change campaigns, with much weaker implementation of more effective population level interventions such as taxation increases and the banning of smoking in public places.^2,3,19–26^ For instance, until recently taxation only accounted for 38% of the price of cigarettes and 9% of bidis in India, which is significantly lower than the WHO recommendation of 70%.^27,28^ The new national government of India has increased tax on cigarettes.^29^ However, there remains a substantial price differential between premium and economy cigarette brands, encouraging product substitution, and bidis continue to be subject to very low taxation. In 2009–2010, one in three employees in India reported being exposed to second hand smoke at their workplace.^20,30^ Simulation of tobacco interventions has shown that 1 million myocardial infarctions (MI) and 0.6 million stroke deaths in India could be averted over the next decade if taxation on cigarettes was increased by 300%.^20^

We found persisting socioeconomic inequalities in tobacco use. This raises a question about whether tobacco control interventions are reaching the most disadvantaged populations. A literature review on the equity dimensions of smoking from developed countries has shown poorer populations to be more sensitive to pricing in tobacco interventions.^31,32^ Little evidence exists that examines this in low and middle income countries like India where the burden of tobacco and socioeconomic disparities in health are areas of concern. Our findings that show declines in bidi use and increase in cigarette use among lower SES groups indicate a potential switch (substitution effect) as cigarettes may have become more affordable and attractive to use compared to bidis.^33–35^ More evidence is needed to examine whether disadvantaged households are becoming “new markets” for cigarette use.

Evidence is also needed to investigate the substantial growth in smokeless tobacco use in India. We found an increase in smokeless tobacco use over time. Smokeless tobacco declined between 2000 and 2005, with not much decline after 2005. This is contrary to expectations of the impact of tobacco control policies introduced after 2004 on smokeless tobacco consumption. This trend may be attributed to the fact that smokeless tobacco products do not receive adequate attention in tobacco control policies in India due to the absence of clear negative externalities as from exposure to second hand smoke from smoked tobacco forms and lower public awareness about health harms of smokeless tobacco.^14,36,37^ The Food Safety and Standards Authority of India (FSSAI) issued a notification prohibiting the use of nicotine and tobacco in food products.^38^ However, the effectiveness of this ban and interventions to ban the production and consumption of smokeless tobacco across Indian states needs to be evaluated for impacts on prevalence and socioeconomic patterning of use.

### Strengths and Limitations

This study used data from repeated cross-sectional surveys (National sample surveys) with comparable data on tobacco consumption over 12 years. These surveys provide nationally representative data on tobacco use along with type and volume consumed. No other survey provides the potential for time-trend comparison.^32^ While the Global Adult Tobacco Surveys (GATS)^30,39^ provides tobacco use data for individuals, it has only been conducted once in India (2009–2010) and hence trends over time cannot be assessed. Consumption information from the National Sample Surveys focusing on households have greater policy relevance as a majority of the economic and healthcare burden of chronic diseases in India is borne by households. Unlike surveys that use information on SES from asset scores, this survey also uses consumption expenditure to assess living standards, which has greater validity in the Indian context.^17^

We acknowledge three main limitations. *First*, data on tobacco from households was self-reported and may suffer from social desirability bias. Since the survey reports prevalence and volume for households instead of individuals, estimates could not be directly compared with other Indian surveys. *Second*, our focus in the present analysis was exclusive use of tobacco products. Only 8% of the households reported use of multiple tobacco products; hence, this focus is unlikely to significantly influence findings. *Third*, the present analysis focused on examining trends for socioeconomic groups and did not investigate causes or determinants of these trends. These need further investigation through more in-depth assessments, focused on specific vulnerable groups. *Finally*, survey data was based on household-level information and did not examine the role of individual determinants, which play a role in the uptake of adverse health behaviors.

## Conclusion

We found persistent SES patterning in tobacco use in India, with some evidence of relative declines in bidi use and relative increases in cigarette use among lower SES households. Substantial increases in smokeless tobacco use were also noted across all groups. These patterns indicate the need for strengthening the enforcement of tobacco control policies and monitoring comprehensive smoke-free legislations. Routine monitoring of inequalities in tobacco use is also required.

## Supplementary Material

Supplementary Tables 1–3 can be found online at http://www.ntr.oxfordjournals.org

## Funding

This article did not receive any specific funding. NB and CM are supported by Wellcome Trust Capacity Strengthening Strategic Award Extension Phase (WT 084754/Z/08/A) to the Public Health Foundation of India and a consortium of UK universities. CM is supported by a NIHR Research Professorship Award.

## Declaration of Interests

*None declared.*

## Supplementary Material

Supplementary Data
