# Supplementary material for: Have Socioeconomic Inequalities in Tobacco Use in India Increased Over Time? Trends From the National Sample Surveys (2000–2012)
Source: Nicotine Tob Res. 2016 Apr 5;18(8):1711–8. doi: 10.1093/ntr/ntw092 (PMC4941603; doi:10.1093/ntr/ntw092)
Supplement: Supplementary Data [file supp_18_8_1711__index.html]

Have Socioeconomic Inequalities in Tobacco Use in India Increased Over Time? Trends From the National Sample Surveys (2000–2012) — Have Socioeconomic Inequalities in Tobacco Use in India Increased Over Time? Trends From the National Sample Surveys (2000–2012) — Supplementary Data 

# Have Socioeconomic Inequalities in Tobacco Use in India Increased Over Time? Trends From the National Sample Surveys (2000–2012)

## Supplementary Data

Data files

- Supplementary Data - Supplementary Data
