# Supplementary material for: Have Socioeconomic Inequalities in Tobacco Use in India Increased Over Time? Trends From the National Sample Surveys (2000–2012)
Source: Nicotine Tob Res. 2016 Apr 5;18(8):1711–8. doi: 10.1093/ntr/ntw092 (PMC4941603; doi:10.1093/ntr/ntw092)
Supplement: Supplementary Data [file supp_ntw092_Webtables.docx]

**Supplementary table 1: Prevalence (%) of any and multiple tobacco use across three National Sample Surveys [1999-2000, 2004-05 and 2011-12] by consumption expenditure, educational attainment and caste/tribe status of households**

| SES Variables | Any Tobacco Use  (95% Confidence Intervals) | | | Multiple Tobacco Use  (95% Confidence Intervals) | | |
| --- | --- | --- | --- | --- | --- | --- |
|  | **1999-2000** | **2004-05** | **2011-12** | **1999-2000** | **2004-05** | **2011-12** |
| *Household Consumption Expenditure Quartiles* |  |  |  |  |  |  |
| Poorest Quartile (Q1) | 61.5 (60.91-62.10) | 49.9 (49.29-50.49) | 62.7 (62.09-63.33) | 7.8 (7.50-8.15) | 5.2 (4.90-5.43) | 10.7 (10.29-11.09) |
| Poorer Quartile (Q2) | 58.9 (58.41-59.56) | 58.6 (58.02-59.17) | 56.1 (55.49-56.79) | 7.9 (7.59-8.22) | 7.9 (7.64-8.27) | 8.8 (8.47-9.21) |
| Richer Quartile (Q3) | 52.3 (51.71-52.83) | 57.9 (57.44-58.54) | 48.3 (47.74- 48.96) | 7.1 (6.86-7.44) | 10.1 (9.77-10.45) | 7.6 (7.24-7.89) |
| Richest Quartile (Q4) | 43.8 (43.27-44.29) | 51.0 (50.51-51.51) | 36.8 (36.30-37.39) | 6.1 (5.81-6.31) | 10.7 (10.41-11.02) | 5.6 (5.36-5.87) |
|  |  |  |  |  |  |  |
| *Highest educational attainment of households* |  |  |  |  |  |  |
| Households with illiterate members only | 61.7 (61.22-62-28) | 62.5 (62.09-62.83) | 59.3 (58.36-60.15) | 8.1 (7.84-8.44) | 9.9 (9.69-10.15) | 9.0 (8.51-9.56) |
| Households with 1 primary schooled member | 60.8 (60.03-61.51) | 49.9 (49.20-50.54) | 63.1 (62.16-64.11) | 8.1 (7.65-8.48) | 7.6 (7.25-7.96) | 11.3 (10.68-11.95) |
| Households with 1 middle schooled member | 57.2 (56.56-57.77) | 44.5 (43.68-45.23) | 59.4 (58.68-60.11) | 7.9 (7.59-8.25) | 6.9 (6.51-7.29) | 10.9 (10.53-11.45) |
| Households with 1 higher secondary schooled member | 45.8 (45.15-46.35) | 32.7 (30.82-34.61) | 51.1 (50.40-51.81) | 6.1 (5.79-6.37) | 3.9 (3.14-4.71) | 7.7 (7.30-8.05) |
| Households with 1 graduate | 31.7 (31.04-32.35) | 29.5 (28.86-30.15) | 37.4 (36.91-37.81) | 4.1 (3.87-4.43) | 4.1 (3.85-4.42) | 5.2 (5.02-5.44) |
|  |  |  |  |  |  |  |
| Household Caste/Tribe |  |  |  |  |  |  |
| Scheduled Tribe (ST) | 69.4 (68.58-70.14) | 71.1 (70.37-71.75) | 67.1 (66.36-67.94) | 10.5 (9.99-11.03) | 12.1 (11.59-12.58) | 14.0 (13.44-14.60) |
| Scheduled Caste (SC) | 63.5 (62.85-64.23) | 62.1 (61.43-62.78) | 59.2 (58.44-59.97) | 8.9 (8.5-9.38) | 10.3 (9.93-10.77) | 10.4 (9.96-10.92) |
| Other Backward Class (OBC) | 54.2 (53.65-54.65) | 53.3 (52.89-53.80) | 49.7 (49.18-50.16) | 7.5 (7.23-7.76) | 7.9 (7.68-8.17) | 7.2 (6.94-7.45) |
| Other Caste (General) | 45.5 (45.12-45.99) | 46.2 (45.69-46.65) | 42.7 (42.14-43.21) | 5.3 (5.09-5.48) | 7.0 (6.78-7.27) | 6.4 (6.10-6.63) |
|  |  |  |  |  |  |  |
| TOTAL | 54.1 (53.86-54.43) | 54.4 (54.09-54.65) | 51.0 (50.71-51.32) | 7.2 (7.09-7.38) | 8.5 (8.34-8.64) | 8.2 (8.01-8.35) |

**Supplementary table 2: Results from pooled Logistic Regression models (Adjusted Odds Ratios (AORs)) for prevalence of use of any and multiple tobacco products by consumption expenditure, educational attainment and caste/tribe status of households (2000-2012)**

|  | Any Tobacco Use | Multiple Tobacco Use |
| --- | --- | --- |
|  | **AOR (SE)** | **AOR (SE)** |
| t_1_ (2005) | 1.06* (0.03) | 1.41*** (0.07) |
| t_2_ (2012) | 1.17*** (0.04) | 1.49*** (0.09) |
|  |  |  |
| Q_1_ (Poorest 25%) | 1.01 (0.02) | 0.70*** (0.02) |
| t_1__ Q_1_ (interaction: 2005 x Poorest 25%) | 0.72*** (0.02) | 0.59*** (0.03) |
| t_2__ Q_1_ (interaction: 2012 x Poorest 25%) | 1.18*** (0.03) | 1.16** (0.06) |
| Q_2_ (Poorer 25%) | 1.09*** (0.02) | 0.83*** (0.03) |
| t_1__ Q_2_ (interaction: 2005 x Poorer 25%) | 0.87*** (0.02) | 0.72*** (0.03) |
| t_2__ Q_2_ (interaction: 2012 x Poorer 25%) | 1.13*** (0.03) | 1.16** (0.05) |
|  |  |  |
| Illiterate households (Households with illiterate members only) | 2.47*** (0.06) | 1.66*** (0.07) |
| t_1__ Illiterate households (interaction: 2005 x illiterate households) | 1.18*** (0.04) | 1.29*** (0.07) |
| t_2__ illiterate households (interaction: 2012 x illiterate households | 0.84*** (0.03) | 0.90 (0.05) |
| Households with 1 Primary schooled member | 2.49*** (0.06) | 1.68*** (0.08) |
| t_1__ Households with 1 primary schooled member (interaction: 2005 x household with primary schooled member) | 0.76*** (0.03) | 0.96 (0.06) |
| t_2__ Households with 1 primary schooled member (interaction: 2012 x households with primary schooled member) | 0.85*** (0.03) | 0.96 (0.06) |
|  |  |  |
| SC households | 1.88*** (0.04) | 1.58*** (0.05) |
| t_1__ SC households (interaction: 2005 x SC households) | 0.94* (0.03) | 0.91 (0.04) |
| t_2__ SC households (interaction: 2012 x SC households) | 0.81*** (0.02) | 0.86** (0.04) |
| ST households | 1.85*** (0.05) | 1.45*** (0.05) |
| t_1__ ST households (interaction: 2005 x ST households) | 0.98 (0.03) | 0.87** (0.04) |
| t_2__ ST households (interaction: 2012 x ST households) | 0.95 (0.03) | 1.17** (0.06) |
|  |  |  |
| Constant | 0.02 (0.00079) | 0.004 (0.00036) |
| Observations | 346614 | 346614 |
| Pseudo R-squared | 0.16 | 0.11 |

Note: Figures in the second row for each indictor are standard errors

Reference: Richest, Postgraduate, Other Caste households in 2000;

*<0.05, **<0.001, ***<0.0001

**Supplementary table 3: Mean volume of exclusive cigarette, bidi and** smokeless **tobacco use (measured in the number of sticks and grams) for tobacco consuming households by consumption expenditure, educational attainment and caste/tribe status of households across three National Sample Surveys [1999-2000, 2004-05 and 2011-12]**

| Socioeconomic Variables | Bidi Use (Exclusive) | | | Cigarette Use (Exclusive) | | | smokeless Use (Exclusive) | | |
| --- | --- | --- | --- | --- | --- | --- | --- | --- | --- |
|  | **1999-2000** | **2004-05** | **2011-12** | **1999-2000** | **2004-05** | **2011-12** | **1999-2000** | **2004-05** | **2011-12** |
| *Household Consumption Expenditure Quartiles* |  |  |  |  |  |  |  |  |  |
| Poorest (Q1) | 74.7(59.5) | 152.4 (190.2) | 53.4 (51.4) | 17.7 (21.7) | 41.4 (57.7) | 8.6 (10.9) | 18.7 (44.9) | 82.5 (148.4) | 31.1 (27.8) |
| Poorer (Q2) | 99.6 (77.7) | 106.8 (87.5) | 80.3 (76.9) | 25.5 (31.1) | 32.2 (41.7) | 12.8 (15.1) | 23.3 (46.8) | 52.3 (91.7) | 41.6 (39.8) |
| Richer (Q3) | 124.4 (139.2) | 93.6 (90.7) | 108.3 (110.1) | 29.4 (33.1) | 30.0 (39.05) | 19.7 (21.8) | 34.2 (68.4) | 45.1 (53.5) | 52.9 (55.2) |
| Richest (Q4) | 183.4 (413.4) | 87.5 (70.7) | 134.0 (129.5) | 49.9 (67.1) | 32.3 (41.3) | 33.4 (40.2) | 62.1 (160.9) | 42.8 (49.4) | 77.9 (111.8) |
|  |  |  |  |  |  |  |  |  |  |
| *Highest educational attainment of households* |  |  |  |  |  |  |  |  |  |
| Households with illiterate members only | 131.4 (158.1) | 112.9 (126.6) | 123.9 (138.7) | 40.6 (53.1) | 29.9 (39.7) | 23.2 (35.9) | 33.1 (96.2) | 59.7 (108.7) | 58.3 (68.6) |
| Households with 1 primary schooled member | 118.6 (396.9) | 94.6 (74.9) | 84.7 (80.7) | 37.7 (64.6) | 27.3 (33.4) | 21.2 (40.3) | 29.8 (93.2) | 48.7 (54.7) | 42.6 (50.6) |
| Households with 1 middle schooled member | 101.8 (89.3) | 92.3 (79.5) | 78.1 (79.2) | 31.9 (42.6) | 32.5 (47.0) | 18.54 (22.5) | 28.5 (57.8) | 45.6 (61.6) | 43.0 (52.8) |
| Households with 1 higher secondary schooled member | 99.4 (87.5) | 89.5 (75.1) | 76.3 (70.3) | 34.9 (51.8) | 40.9 (47.7) | 21.7 (26.7) | 24.6 (51.6) | 70.4 (65.5) | 38.1 (32.6) |
| Households with 1 graduate | 92.4 (91.1) | 83.9 (65.4) | 70.1 (65.5) | 41.3 (51.9) | 39.8 (48.4) | 24.9 (32.4) | 31.3 (75.2) | 42.0 (48.0) | 40.7 (65.1) |
|  |  |  |  |  |  |  |  |  |  |
| *Household Caste/tribe status* |  |  |  |  |  |  |  |  |  |
| Scheduled Tribe(ST) | 102.7 (149.3) | 96.2 (34.7) | 75.6 (83.7) | 23.7 (38.3) | 22.7 (34.7) | 16.5 (25.8) | 27.4 (93.7) | 54.7 (64.8) | 39.3 (44.8) |
| Scheduled Caste(SC) | 121.9 (348.9) | 106.7 (37.0) | 89.0 (95.0) | 31.3 (61.9) | 28.9 (37.0) | 18.5 (26.1) | 28.3 (58.9) | 58.5 (142.9) | 45.8 (52.9) |
| Other Backward Class (OBC) | 113.5 (131.8) | 111.3 (38.9) | 90.9 (101.2) | 37.2 (50.7) | 31.2 (38.9) | 23.5 (32.9) | 29.2 (92.5) | 51.9 (67.7) | 43.9 (57.1) |
| General Caste | 118.1 (118.6) | 108.3 (47.1) | 88.8 (92.0) | 39.1 (50.3) | 35.6 (35.6) | 24.7 (30.0) | 34.5 (71.8) | 62.0 (104.2) | 49.6 (65.9) |
